# The Orexin/Hypocretin System, the Peptidergic Regulator of Vigilance, Orchestrates Adaptation to Stress

**DOI:** 10.3390/biomedicines12020448

**Published:** 2024-02-17

**Authors:** Miklós Jászberényi, Balázs Thurzó, Zsolt Bagosi, László Vécsei, Masaru Tanaka

**Affiliations:** 1 Department of Pathophysiology, University of Szeged, H-6701 Szeged, Hungary; miklos.jaszberenyi@med.u-szeged.hu (M.J.); bazska82@gmail.com (B.T.); bagosi.zsolt@med.u-szeged.hu (Z.B.); 2 Emergency Patient Care Unit, Albert Szent-Györgyi Health Centre, University of Szeged, H-6725 Szeged, Hungary; 3 Department of Neurology, Albert Szent-Györgyi Medical School, University of Szeged, H-6725 Szeged, Hungary; vecsei.laszlo@med.u-szeged.hu; 4 HUN-REN-SZTE Neuroscience Research Group, Hungarian Research Network, University of Szeged (HUN-REN-SZTE), Danube Neuroscience Research Laboratory, Tisza Lajos krt. 113, H-6725 Szeged, Hungary

**Keywords:** orexins, neuropeptides, neurotransmitters, stress, feeding, temperature regulation, fear, anxiety, learning, sleep–wake disorders

## Abstract

The orexin/hypocretin neuropeptide family has emerged as a focal point of neuroscientific research following the discovery that this family plays a crucial role in a variety of physiological and behavioral processes. These neuropeptides serve as powerful neuromodulators, intricately shaping autonomic, endocrine, and behavioral responses across species. Notably, they serve as master regulators of vigilance and stress responses; however, their roles in food intake, metabolism, and thermoregulation appear complementary and warrant further investigation. This narrative review provides a journey through the evolution of our understanding of the orexin system, from its initial discovery to the promising progress made in developing orexin derivatives. It goes beyond conventional boundaries, striving to synthesize the multifaceted activities of orexins. Special emphasis is placed on domains such as stress response, fear, anxiety, and learning, in which the authors have contributed to the literature with original publications. This paper also overviews the advancement of orexin pharmacology, which has already yielded some promising successes, particularly in the treatment of sleep disorders.

## 1. Introduction: Neuropeptides as the Modulators of the Connectome

In Section 1 and Section 2, a general overview of the orexin system is given, and Section 3 and Section 4 will be devoted to those fields in which the authors have considerably contributed to the literature. Neuropeptide research started more than a hundred years ago with the discovery of the effects of vasopressin [1] and oxytocin [2] and tissue extraction of the first classical neuropeptide: substance P [3]. Since then, more and more distinct features of neuropeptides have been identified that clearly separate them from classical neurotransmitters [4,5,6]. First, some obvious biochemical features justify the differentiation. They are much larger molecules than classical neurotransmitters; therefore, the energy requirements for their synthesis and transport well exceed those of these neurotransmitters. Functionally, their release is not confined to the synapses, although some portions of their pool are also frequently co-secreted together with classic neurotransmitters. In contrast, they can be released from the dense core vesicles of practically any portion of the neurons [4,5,6]. Unlike classic neurotransmitters, neuropeptides are not taken back economically by a reuptake system but are metabolized by peptidases. Nonetheless, this process frequently yields biologically active compounds [6]. Further, due to their prolonged half-life, neuropeptides can diffuse to long distances; therefore, they act not only post- and pre-synaptically (i.e., in a paracrine and autocrine fashion) but also in an endocrine manner [6] through the operation of G-protein-coupled receptors (GPCRs) [6]. Due to these characteristics, their effects develop slowly but are usually longer lasting. Their impact, compared to that of classic small molecular and even gaseous neurotransmitters, is almost always less robust, which strongly resembles the activity of hormones [7]. That is why in the literature some members are still referred to as neurohormones and their activity has been formulated as “neuromodulation” [4,5,6], which represents a “mild” or “functionally buffered” form of signal transduction [4,5,6]. A unique feature of neuromodulation is that it is realized using a much broader arsenal of receptors than that of neurotransmitters [5]. Also, the ligands themselves show immense structural versatility because, in some families, almost infinite splice variants can be produced from several copies of an ancestral gene [5]. Therefore, it is not surprising that neuroscience has struggled to formulate a rigid functional definition of neuromodulation, in contrast with that of neurotransmission and neurotransmitters [8].

Accordingly, neuropeptides appear to represent an individual and separate form of transfer of biological information, somewhere in between that of the classical neurotransmitters and peripheral hormones. The secretion of these modulators gives rise to less acute but, in the long run, more profound changes in the neural connectome, the operative framework of neurophysiology [9]. The multi-faceted activity and redundancy of these signaling molecules provide an indispensable component of the plasticity and resilience of the central nervous system (CNS). This is further augmented by the bewildering diversity and extremely broad distribution of several neuropeptide families. Certain groups and their receptors are expressed at every level of neuroendocrine control [4,5,6].

It is apparent that the challenges to which the CNS is exposed ultimately will give rise to changes in translational processes. These will manifest themselves in the modification of receptor and enzyme expression, synaptic plasticity, and the dendritic pattern and structure and finally the complete wiring of the connectome [9,10]. Neuropeptides represent an essential but so-far overlooked element of the translational machinery, bridging the gap between volatile functional alterations and permanent structural changes. As peptides, they represent one of the earliest steps (along with neurotransmission-evoked peptide and protein synthesis) in the translational processes of information signaling in the CNS. Their modulatory action via extremely versatile ligands, the corresponding receptors, and a multitude of stimulated signaling cascades make them the most flexible line of neuroendocrine plasticity and adaptation [4,5,6].

The experiments carried out on the orexin/hypocretin system especially support this view: its cooperation with other neuropeptides appears to harmonize the autonomic, behavioral, and endocrine response to arousal [11,12,13,14,15,16,17,18,19,20]. Hence, firstly, the present paper gives a general overview of the general biochemical, anatomical–histological, physiological, and pathophysiological features of the orexin/hypocretin system. This review focuses on those specific fields (stress response, thermoregulation, fear, anxiety, and learning) in which the authors have also contributed to the literature.

## 2. The Hypocretin/Orexin Peptide and Receptor Family

**Figure 1:**
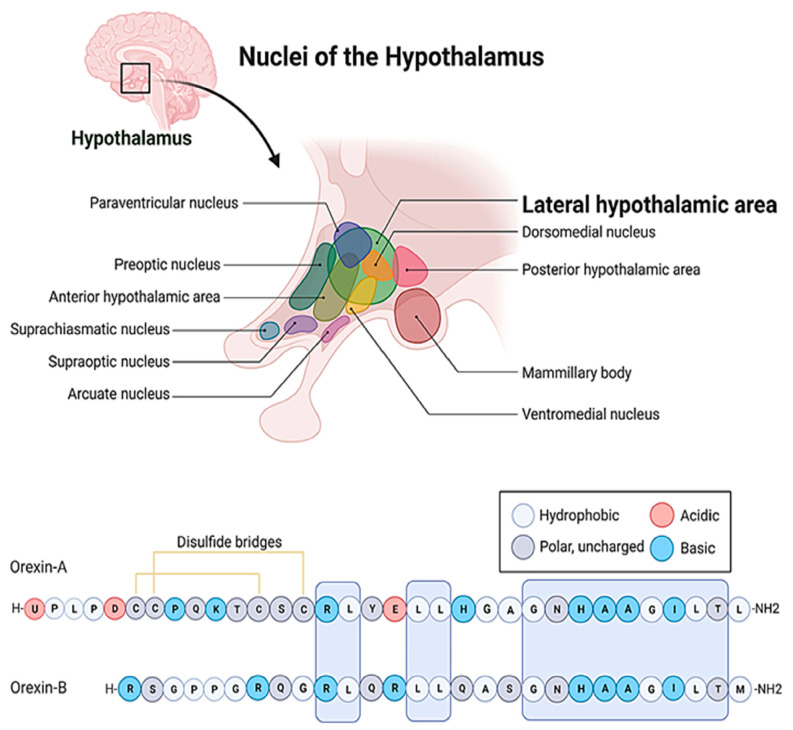
The localization of the lateral hypothalamic area and the amino acid sequences of the orexin/hypocretin family peptides. The letters stand for the one letter code of amino acids. A: Alanine, C: Cysteine, D: Aspartic acid, E: Glutamic acid, G: Glycine, H: Histidine, I: Isoleucine, K: Lysine, L: Leucine, M: Methionine, N: Asparagine, P: Proline, Q: Glutamine, R: Arginine, S: Serine, T: Threonine, U: Pyroglutamic acid, Y: Tyrosine.

The hypocretin/orexin system represents an extremely complex neuropeptide network in the CNS [21,22]. The seminal papers [23,24,25] that dealt with the discovery of these ligands and their receptors also demonstrated the hyperphagic [23] and neuroexcitatory activity [24] of orexins and the unique distribution pattern of the system. The orexin/hypocretin system, similarly to melanin-concentrating hormone (MCH)-positive neurons [26], has a well-circumscribed expression in the hypothalamus (Figure 1) [24,27]. Its cell bodies are restricted to the lateral, dorsal, dorsomedial, and perifornical areas, and the whole population does not exceed 50,000–80,000 cells in the hypothalamus. However, its axon terminals reach distant regions, and its receptors are scattered throughout the whole CNS [25].

At the cellular level, so far, two ligands (orexin-A, orexin-B) and two receptors (OX1R and OX2R) of the system have been described (Figure 1) [23,24]. The peptides biochemically belong to the incretin family, but they bear weak structural resemblance only to a few members of the group [23,24]. Even orexin-A and orexin-B differ by 50% of their primary structure. Both peptides are cleft from pre-pro-orexin (PPO) and are amidated C-terminally, but orexin-A is larger, comprising 33 amino acids, while orexin-B consists of only 28 residues [28]. Orexin-A is also less prone to proteolytic degradation because it comprises an N-terminal pyroglutamate residue and two disulfide bonds. Additionally, orexin-A is more hydrophobic, and therefore it can bypass more efficiently the blood–brain barrier (BBB) [29]. These orexins also exhibit significantly different receptor affinities [23,24,30], which is definitely attributed to the fact that the orexin receptors (OXRs) share only 64% amino acid identity [28,31]. The two receptor subtypes create diversity within the cellular signaling pathways [28,31,32,33,34]. Both OX1R and OX2R activity is mediated by Gq_11_, which, in turn, leads to the activation of phospholipase C (PLC), phospholipase A (PLA), and phospholipase D, ultimately resulting in an increase in cytosolic Ca^2+^ and the activation of protein kinase C (PKC). In addition, OX1R can elevate the intracellular Ca^2+^ level by activating non-selective cation channels (NSCCs) [31]. On the other hand, OX2R can also inhibit adenyl cyclase (AC) and protein kinase A (PKA) through the G-protein-coupled pathway. The potential dimerization of the OXRs and the structural overlap between OXRs and some other GPCRs lend further diversity to the signal transduction of the system [31]. For example, certain neuropeptide receptors, such as the type-2 neuropeptide-Y (NPY) receptor, the thyrotropin-releasing hormone (TRH) receptor, the cholecystokinin (CCK) type-A receptor, and the NK2 neurokinin receptor, show some similarities (26%, 25%, 23%, and 20% identity, respectively) to the orexin receptors [23]. The highest structural similarity is exhibited by the neuropeptide FF (NPFF) receptor of the RF-amide peptide family, which is 37% identical to OX1R and 35% identical to OX2R, respectively [35,36].

Neither the distribution of the immunoreactivity of the two orexins [37,38] nor the expression of OX1R [30,39,40,41] and OX2R [30,40,41,42] completely overlaps. This, together with the aforementioned distinct features of the pharmacokinetics of orexin peptides and the differences in the signal transduction of OX1R and OX2R [32,33,34], must be responsible for some divergence in the physiologic and pathophysiologic actions of orexin-A and orexin-B [38].

## 3. The Orexin System, as an Indispensable Regulator of Arousal, Cooperates with the Central Oscillator to Control Circadian Activities

At the systemic level, the function of target areas of the orexin neurons has suggested numerous clues on the feasible actions of orexins [25] (Table 1). Although, at first, orexins were proved to play an important role in hedonic feeding [23,43], later publications unveiled that the most important aspect of their functional spectrum could be the regulation of arousal [44]. Later studies also verified that the orexin network receives important input from the neurons of the circadian system [45,46], the most important center of which is the suprachiasmatic nucleus (SCN). These results substantiated the way circadian rhythms and arousal are synchronized in the mammalian brain. The mammalian circadian clock itself is hierarchically organized [47,48,49,50]. Its main components are the input signaling pathways, the main pacemaker, and the output signaling pathways [47,48,49,50]. The most important inputs to the SCN are photic stimuli, which arrive from the retina through the retinohypothalamic pathway [47,48,51]. The SCN serves as the “master clock” for the brain and the body and controls the activity of “local clocks”. In the CNS, its outputs reach several autonomic centers in the hypothalamus, such as the ventrolateral preoptic nucleus (VLPO), the arcuate nucleus (ARC), the organum vasculosum laminae terminalis (OVLT), the median preoptic area (MnPO), the lateral hypothalamus, the medial preoptic area (MPO), and the paraventricular nucleus (PVN) [48]. Therefore, the central oscillator determines the circadian, diurnal, or mensual characteristics of the sleep–wake cycle, food and fluid intake, core temperature, vigilance, and several endocrine activities, such as the estrous cycle and the activity of the hypothalamic–pituitary–adrenal cortex (HPA). The SCN also targets several extrahypothalamic centers from the brainstem, through the pineal gland to the hippocampus [47]. This way, it also provides temporal clues on the entrainment of arousal, autonomic control, pain sensation, and even higher cortical activities such as mood, affection, and learning. The connection between the SCN and the orexin neurons has two-way bidirectional components, as described in recent publications [48]. However, a strong endocrine connection has also been verified between the two centers, through the melatonin secretion of the pineal gland [51]. Ultimately, indirect communication is established between the central oscillator and the orexin neurons at the level of the ascending reticular activation system (ARAS). First, the ARAS is undoubtedly the most important output of the lateral hypothalamus since orexinergic cells target several important centers of the ARAS, such as the pedunculopontine tegmental (PPT) and lateral dorsal tegmental (LDT) nuclei in the mesopontine tegmentum (MT), the nucleus raphe (NR), the locus coeruleus (LC), and the periaqueductal gray (PG) [25,52]. Second, these nuclei also provide the most important non-photic inputs to the central oscillator [48].

The nuclei of the ARAS operate with the classical neurotransmitters (acetylcholine, serotonin, norepinephrine, and dopamine, respectively) and foster the inputs to the dualistic centers of sleep–wakefulness regulation: the tuberomammillary nucleus (TMN) and the VLPO of the hypothalamus. The histaminergic TMN and the galanin- and γ-amino-butyric-acid (GABA)-positive VLPO control sleep onset according to a flip-flop mechanism [53,54,55]. It seems that the orexin-positive cells facilitate arousal through indirect disinhibition: they block the GABAergic output of the VLPO through the stimulation of the above-mentioned cholinergic and monoaminergic nuclei of the ARAS [25,56,57,58,59,60,61]. In this activity, OX2R mediation appears to play a predominant role. OX2R antagonism is sufficient to induce sleep, while OX1R blockade even attenuates this phenomenon [62,63,64]. This way, the orexin system could be easily categorized not only as the master regulator of arousal [65] but also as a significant modulator of the sleep–wake cycle and other circadian rhythms [44,53,66,67]. The indispensable role of the orexin system in the maintenance of vigilance is strongly supported by the finding that narcolepsy and cataplexy observed in dogs [68] and mice [69] can be solely attributed to the deficiency of the orexin/hypocretin system. Moreover, by now, it has been supported by several observations that human cases of narcolepsy with cataplexy [70,71,72] can also be attributed to either the abnormal development [73] or acquired immunological destruction [74] of the orexin–hypocretin system. Narcolepsy is characterized by REM intrusion into wakefulness, and REM sleep depends on the cholinergic activity of the ARAS, which is gated by the histaminergic neurons of the system [60,61,64]. These antagonistic centers both receive orexinergic input [25]. However, it appears that it is the selective deficiency of histaminergic gating that is responsible for the disease. This can be attributed to the absence of OX2R in the histaminergic neurons [60,61,64]. These observations offer great therapeutic opportunities not only for sleep disorders but also for abnormalities of these physiological functions, which are influenced by the orexin/hypocretin network. The most important physiological processes the circadian control of which the orexin system modulates are food and fluid intake [75,76,77], metabolism and thermoregulation [78], the activity of the HPA axis [11,13,65,79,80,81,82], and reproduction [83,84]. As mentioned above, the anatomical connections, which provide the foundation of these physiological actions, have also been verified: the orexinergic axons target the ARC, the PVN, and the preoptic and supraoptic nuclei (PON and SON, respectively) of the hypothalamus [10,53].

Even the initial publications suggested that orexins increase body weight and acutely stimulate food intake [23,85]. Due to the hyperphagic activity and specific localization of the orexin neurons, at least a group of their population can certainly be identified with some portion of the classic glucose-sensitive feeding center of the ventrolateral hypothalamus [86,87,88,89,90]. The orexin-positive neurons are bidirectionally connected to the ARC and the PVN, which operate in a well-known dualistic manner in food intake regulation. The most important stimulatory neuropeptides are NPY and agouti-related peptide (AgRP) in the ARC and TRH in the PVN. The inhibitory neurohormones are cocaine- and amphetamine-regulated transcript (CART) and melanocortins (MCs) in the ARC and CRH in the PVN [91,92]. According to the literature, orexin-A evokes the activation [93] of the OX1Rs [77] in the ARC, and the PVN mediates the hyperphagic effect of the system. However, the role of orexins is more complex since, in the long run, it is their deficiency that is associated with weight gain [94], which is attributed to their two-pronged action in thermoregulation. The anatomical substrate of this activity is the connection of the orexin system to the PON and the dorsomedial hypothalamus (DMH) [25]. The orexins concurrently activate heat loss and thermogenesis. That is why they can either decrease or increase the core temperature depending on the experimental settings [12,95,96,97]. In summary, they stimulate heat dissipation [12,97] through sympathetic vasodilation [95], which is mediated by OX1R. However, it is accompanied by the modulation of metabolism in the brown adipose tissue (BAT) [98]. In the sympathetic nervous system, OX1Rs apparently activate [96,99] while OX2Rs inhibit [100] non-shivering thermogenesis in the BAT. Obviously, concomitant increases in heat generation and heat dissipation prevent the excessive accumulation of fat.

Orexins also modulate the activity of the reproductive axis [83,84,101,102]. This action is bidirectional and brain-region- and, in females, estrous-cycle-dependent. Further, both orexin-A and orexin-B and both OXR1 and OXR2 take part in the control of the hypothalamic–pituitary–gonadal (HPG) axis [103]. The orexin system may supply period-dependent inputs to the HPG axis. Moreover, it can provide the link between self-preservation and species preservation since a well-fed but not overweight subject can optimally guarantee the survival of its offspring. It appears, that in the regulation of the HPG axis, the interaction between the orexigenic and anorexigenic (such as leptin) peptides plays a pivotal role [104,105]. As far as self-preservation is concerned, orexins have also been proved to be one of the most important orchestrators of the stress response [80,81]. Further, they modulate all threat-related adaptive behavioral processes [106,107,108], even fear-related learning [109,110,111,112].

**Table 1:** An outline of the orexin/hypocretin connectome in physiologic regulation [113].

Input Region	Core Region	Target Region	Receptor	Function
Thalamus, TMN, SCN	PFA, LHA	Thalamus, LC, DR, VTA, TMN	OX2R	Circadian regulation, arousal, wakefulness [44,53,65,66,67]
Peripheral signals, ARC, PVN, SCN	LHA, DMH	VMH, ARC, PVN, NAc	OX1R	Food intake [23,43,85]
Peripheral receptors, brainstem, septum	LHA, PFA	PAG, NST, PON, PVN, RVLM, RVMM, VTA	OX1R, OX2R	Autonomic regulation: thermoregulation [12,95,96,97], cardiovascular responses [114,115]
Thalamus, hippocampus, PVN, BNST	PFA, DMH	CeA, LA, LC, PPT, PVT, BNST and MTL	OX1R	Emotions (anxiety, fear, mood) [109,110,116]
Thalamus, hippocampus, SCN	LHA, DMH	VTA, NAc, DR, IC, and PFC	OX1R, OX2R	Cognition, reward, and addiction [117,118]
Pituitary, adrenal gland, thalamus, brainstem, SCN	LHA, DMH	PVN, PON	OX1R, OX2R	GAS [11,65,119] and fight-or-flight response [80,81]
Pituitary, ovary, brainstem, SCN	LHA, DMH	ARC	OX1R, OX2R	Gonadal functions [83,103]

ARC: arcuate nucleus, CeA: central amygdala, DMH: dorsomedial hypothalamus, BNST: bed nucleus of stria terminals, DR: dorsal raphe, IC: insular cortex, LA: lateral amygdala, LC: locus coeruleus, LHA: lateral hypothalamic area, MTL: medial temporal lobe, NAc: nucleus accumbens, NST: nucleus of the solitary tract, OX1R: orexin-1 receptor, OX2R: orexin-2 receptor, PAG: periaqueductal gray material, PFA: perifornical area, PFC: prefrontal cortex, PON: preoptic nucleus, PVN: paraventricular nucleus, PPT: pedunculopontine tegmental nucleus, RVLM: rostral ventrolateral medulla, RVMM: rostral ventromedial medulla, TMN: tuberomammillary nucleus, VMH: ventromedial hypothalamus, VTA: ventral tegmental area.

However, the orexin system has been proven to act as an important regulator even in physiological and pathophysiological processes which possess less obvious temporal characteristics: to mention a few of them, pain sensation [120,121], anxiety, mood, reward processes, and addiction [21,79,106,122,123]. The orexin system represents the poster child of neuropeptide regulation: its perikarya are confined to a small region, but it signals diverse evolutionarily conserved functions to distant targets. In summary, we can say that its principal role must be the temporal gating of brainstem functions [10,53].

## 4. The Role of Orexins in the Regulation of the Stress Response

The reaction of our neuroendocrine regulation to adverse challenges is provided by the interaction between the sympathoadrenal (SA) system and the HPA axis [124]. Although they represent two distinct pathways, the line between them is frequently blurred, even in scientific literature. Perhaps this is due to their interwoven functions, as they complement each other’s activity while trying to maintain the homeostatic balance of challenged individuals. However, the SA response described by Cannon [125] is carried out according to the cooperation of the autonomic nervous system and the adrenal medulla, while the stress response, discovered by Selye [126], relies on the reaction of the HPA system, one of the central neuroendocrine axes later described by Schally, Guillemin [127], and Vale [128]. Unfortunately, by now, the terminology has been oversimplified, and stress response (though it has several stages) is frequently used as an umbrella term for both responses. Only in meticulous descriptions are these two neuroendocrine reactions clearly separated. To avoid confusion, for the HPA response, the most suitable term is the synonym (general adaptation syndrome: GAS) coined later by Selye [129]. Nonetheless, the distinction between the two pathways is of crucial importance because it helps clarify many contradictions in the literature. Some conflicting responses to certain stress paradigms could be easily resolved by clear discrimination between the two potential targets of adverse stimuli, that is, the SA system and the HPA axis.

It is well known that many neuropeptides modulate the activity of the HPA axis. For instance, NPY, neurotensin (NT), ghrelin, apelin, and endomorphins activate [14,17,20,130,131,132,133] while oxytocin and natriuretic peptides inhibit the system [134,135,136,137]. The output of the HPA axis is quite uniform: it begins with the pituitary translation and cleavage of pro-opiomelanocortin (POMC), yielding adrenocorticotropic hormone (ACTH), which, upon secretion, stimulates glucocorticoid release from the adrenal cortex [126,129]. However, in sharp contrast with the output, the input of the HPA axis is extremely diverse and involves a multitude of neuropeptides in signal transduction [131]. Therefore, it is not surprising that the modality (systemic or neurogenic) and schedule (acute, repeated, or chronic) of the stressors strongly influence the extent of the HPA response [138]. Systemic challenges (e.g. osmotic, immune, etc.) perturb the homeostatic balance of the organism, which is directly projected to the brainstem, while neurogenic paradigms (fear, pain) are processed by the cerebral centers [124]. The responses to these two types of challenges are signaled in a dichotomized manner in the brain. The corticotrope-releasing hormone (CRH)-positive neurons of the PVN are responsible for the acute and processed stimuli, while parvocellular arginine vasopressin (AVP) cells in the PVN and the SON maintain responsiveness to chronic, repeated, and homeostatic challenges [139]. It is also worth noting that neuropeptide modulation perfectly complements the built-in brakes of the GAS: the stepwise ultrashort, short, and long loop feedback mechanisms provided by CRH, ACTH, and the glucocorticoids, as well as the potent anti-inflammatory action of the glucocorticoids [124]. These mechanisms are called stress coping or stress resilience, and they harness the severe inflammatory response (SIRS), which otherwise could consume the organism [124,140,141].

As far as the effect of orexins on the HPA axis and the SA system is concerned, the two responses work hand in hand. Namely, in both responses, orexins play a predominantly stimulatory role [80,81]. However, according to the data from the literature, they are stimulated separately. It seems that the SA system is uniformly activated by orexin-A, which stimulates the OX1Rs expressed in the neurons of the nucleus of the solitary tract (NST), the LC, and the sympathetic neurons [53,65,75,80,142,143,144]. Ultimately, it is not a far-fetched idea to state that the perifornical, dorsal, dorsomedial, and lateral hypothalamic foci of orexin-positive neurons can be identified with those in the caudal hypothalamic region, which were demonstrated to be essential for an intact “fight or flight” and “sham rage” response by Philip Bard and Walter Hess [8,106].

However, as for the HPA axis, the picture is more complex. Soon after the discovery of the dense orexinergic innervation of the hypothalamic centers (PVN and SON) of the GAS, the scientific rivalry surrounding this highly coveted topic begot several important papers, which established that orexin neurons can activate the HPA axis predominantly at the hypothalamic level [11,65,119]. The main targets of the orexin neurons are the OX2Rs [145] expressed in the CRH-positive perikarya of the PVN [25,119]. Nonetheless, later publications showed that the connection between the orexin- and CRH-positive neuron population is bidirectional since abundant CRH-positive fibers land in the orexinergic perikarya of the hypothalamus [146,147,148]. Apparently, orexin-evoked HPA activation also involves the release of noradrenaline and NPY [13,149,150,151], which can significantly diversify its processing [140,141].

As far as the input of the HPA axis is concerned, the activity of orexins appears to be stressor- and schedule-specific [80,81]. In an acute setting, the challenges processed with heightened arousal (aversive odors, novelty, and contextual fear) give rise to more conspicuous activation of the orexin neurons (verified according to *c-fos* expression) than systemic challenges (e.g., cold exposure) or long-lasting procedures (e.g., restraint and immobilization) [80,81,108,152]. Nevertheless, while acute stress mostly activates the orexin neurons, experiments with chronic or repeated stressors returned mixed results [80,81], the findings of which may reflect an adaptation to unavoidable and permanent challenges. Further studies have revealed that the involvement of the orexins in the GAS depends on not only the modalities and schedule of the applied stressor but also the species and gender of the investigated subjects. Females and strains with better stress resilience phenotypes release less orexin in response to adverse stimuli [80,81].

Regarding the output of the HPA axis, orexins have been proven to stimulate the HPA axis not only at the hypothalamic but also at the pituitary and adrenal levels [80,81]. This finding is of crucial importance as peripheral activation stabilizes the HPA response to prolonged stimuli. It nurtures sufficient basal activity but also prevents an exaggerated hypothalamic response by maintaining negative feedback through the release of ACTH and glucocorticoids. Apparently, the orexin/hypocretin system also plays a crucial role in the cooperation and seamless integration of the GAS and the “fight or flight” response. Even the earliest publications which dealt with the orexin system demonstrated the dense innervation of the BNST, a limbic center, which harmonizes the activity of the SA system and the GAS [25]. Therefore, it is not unrealistic at all to conceive of orexins as the coordinators of the stress response to challenges with heightened arousal [80,81].

## 5. The Role of the Orexins in the Regulation of Anxiety and Reward-Related Learning Processes

There is a consensus in the scientific community that the orexin system is the most important peptidergic mediator of the ARAS [44,53,65] and thus arousal processes. Its indispensable role in the regulation of vigilance was ultimately confirmed by the observation that its deficit leads to irreversible functional consequences both in congenital and acquired disorders of arousal and sleep: narcolepsy and cataplexy [68,70,71,72,73]. However, over time, it became obvious that not only the maintenance of wakefulness but also the fine-tuning of arousal-related behavior belongs to the functional repertoire of the orexin system [109]. This concept is supported by the experimental data, which have demonstrated that orexins stimulate attention, rearing, and locomotion as well as such anxiety-related stereotyped behaviors as grooming and freezing [44,53,65,153,154,155,156]. Further experiments are needed to verify its role in such ancient behavioral patterns as thanatosis [157,158].

It is well known that threats are the strongest activators of vigilance. They evoke alertness and attention and then provoke an emotional response, that is, fear. And fear has a huge impact on all aspects of behavior, which manifests itself in anxiety and alterations in mood and cognition, among other things [159]. This strong association considered, it is not surprising that the appropriate behavioral responses to both transient and permanent threats are accompanied by the stimulation of the orexin system, one of the key components of arousal regulation [160]. It seems that in the central processing of threats, first, the robust stimulation of the ARAS involves activation of the orexinergic network [44,53,65]. In turn, its hypothalamic foci fine-tune the neurotransmitter release [107,111,112,116,160,161] of those brainstem and limbic centers which are responsible for the regulation of emotions, affections, mood, and learning processes [80,160,162]. The neuroanatomical substrate of fear- and reward-related learning consists of three components: first, the sensory center, the thalamus, and second, the primary modulator, the amygdala. However, the third, the output, is modality-dependent. Reward-related learning is controlled by the ventral striatum and the ventral tegmental area (VTA), while it is the medial temporal lobe (MTL) that synchronizes fear-related learning [159,163]. Both the central (CeA) and the extended amygdala (e.g., BNST) take part in the facilitation of fear-related memory engraving. Then, contextual memory consolidation is achieved through the activity of such components of the MTL as the hippocampus and the entorhinal, perirhinal, and parahippocampal cortices [163]. On the other hand, reward-related memory is processed by the basolateral amygdala, and the output reaches the prefrontal cortex (PFC). This connection, however, is permanently fine-tuned by such mesolimbic reward centers as the VTA and the nucleus accumbens (NAc) [164]. The orexin-induced activation, similarly to the neuroendocrine parallels, is bidirectional, as inputs from the limbic structures (the septum, BNST, basal forebrain, central amygdala, and hippocampus) account for most of the telencephalic inputs to the orexin neurons [165]. The activity of this connection was demonstrated in a multitude of experiments, which revealed that anxiogenic stimuli, such as exposure to cat odor or novelty [106,107,108,166], gave rise to the activation of the orexin network. In this way, it is not surprising that orexin treatment enhances the startle response [167] and passive avoidance [112]. Later studies specified that the function of the amygdala, which monitors emotional learning and arousal-driven memory consolidation [168], is controlled by the orexin network indirectly and directly. The amygdala receives direct orexinergic projections [25], but the indirect pathway (through the LC) is more important: it carries rich noradrenergic projections to the lateral amygdala [160]. These studies ultimately revealed that orexins play an especially important role in cue-dependent fear memory formation, mainly indirectly through the release of noradrenaline in the LC [109,110,111]. Both this indirect pathway and the direct pathway utilize OX1Rs. The LC expresses exclusively OX1Rs [169], and the direct pathway targets the OX1Rs in the lateral nucleus of the amygdala and hippocampus [160,170]. This hypothesis was confirmed by the finding that orexin-A, which prefers OX1R, proved to promote emotional learning, memory consolidation, and retrieval processes in a passive avoidance paradigm and in social learning [112,171]. Hence, facilitation of learning ensures the avoidance of potentially harmful events, which ultimately is preventive and therefore the most effective technique in stress coping.

Recently, more and more attention has been paid to specific aspects of orexin-mediated behavioral responses: reward and addiction [117]. Functionally, orexins are prime examples of hedonistic neuropeptides [172]. In the past two decades, orexins have been proven to take part in the control of such strong natural rewards as food and fluid intake [23,43,75,76,77] and reproduction [83,102]. They especially stimulate binge eating of palatable food [85,173]. These physiological data have already been substantiated by the histological findings, as well. The cells of the two principal dopaminergic pathways (mesolimbic and mesocortical tracts) of the reward system [8] bear orexin-positive boutons [25]. The beginning (the VTA) of the pathways, the relay centers (the BNST, the CeA, and the hippocampus), and the endings (the NAc, the target of the mesolimbic tract, and the PFC, the target of the mesocortical fibers) receive rich orexinergic inputs [25]. Perhaps the orexinergic inputs can fine-tune the SCN-independent circadian activity of the reward system [118]. However, this hypothesis requires experimental verification.

In addition to these primary hedonistic features, orexins appear to control stress resilience, especially in chronic and repeated experimental conditions [80,81]. This concept was reinforced by the histological data when the interaction between the orexin network and another hedonistic peptide, ghrelin, was verified [89,174,175]. It was suggested that they should augment each other’s activity in stress coping, which could dampen the detrimental psychological consequences of adverse stimuli. Therefore, it was postulated early on that they could take part in the mediation and modulation of behavioral responses evoked by not only natural but also pharmacological rewards. Since then, numerous publications have revealed that the orexin connectomics shows significant alterations not only in obesity [89,174,175] but also in drug dependence [176]. This way, several pathophysiological responses in addiction, such as reinforcement, drug-seeking, and self-administration, were attributed to its modified activity [177]. The orexin network is unambiguously upregulated in cocaine [178,179,180] and opiate abuse [181,182,183], while in the case of other substances, the reaction is more complex [177]. Acute alcohol consumption increased while chronic alcohol, Δ^19^-tetrahydrocannabinol, and nicotine abuse decreased the orexin expression in the hypothalamus [177,184,185,186]. Acute changes must be related to the direct effect of the addictive substance. However, chronic changes may reflect the response to a specific stress paradigm: drug withdrawal. Ultimately, these schedule-dependent bimodal changes also appear to reflect adaptation, a form of stress coping.

## 6. The Cooperation between Orexinergic and Other Peptidergic Neuronal Networks

As has been discussed, the somas of the orexin/hypocretin neurons are restricted to the caudal portion of the hypothalamus [23] but their axon terminals reach distant regions, and their receptors are scattered throughout the whole CNS [25]. This spatial concentration of the cell bodies is not unique in the CNS since it can be observed among other neuropeptides such as MCH [187], ghrelin [188,189], and neuromedin-S [190]. As is the case with other neuropeptides, the feasible interaction of the orexin system and other networks greatly broadens the regulatory repertoire of the orexin/hypocretin neurons. The potential partners are MCH- [191], NPY- [192], apelin- [14,193,194,195], ghrelin- [15,16,17,196], and neuromedin-positive [20,197] networks, which show a marked functional overlap with orexins in the regulation of food intake, the sleep–wake cycle, stress response, and behavior. These networks may cooperate with each other, but the intact function of the orexin system is required for normal processing of arousal-related processes [68,69,70,71,72].

So far, besides releasing hormones of the hypothalamic–pituitary–target organ axis, only the orexin system has proven to be an essential neuropeptide in the regulation of the CNS. However, the interpolations of other neuropeptides and neurotransmitters in the signal transduction of the orexin neurons lend immense diversity and flexibility to the orexin-regulated responses [83]. This is because the neuropeptide ligand and receptor families typically consist of several members, which may have numerous splice variants, can also be modified by peptidases after secretion, and can act on an arsenal of receptors [4,5,6,131,140,198]. With the different binding affinities and activities taken into consideration, the number of potential interactions between this abundance of ligands and receptors is infinite and may span from full agonism to complete antagonism [4,5,7,198].

Cooperation both in the afferent and efferent pathways of the orexin system has been verified. In the input, monoamines [25], NPY [199,200], and ghrelin [89,174,175] seem to play the most important role, while in the output, NPY [12,13,151,199,201], POMC [202], and also monoamines [25,107,112,161,203] have been identified. Unambiguously, the pathways between the LC and the lateral hypothalamus are the most important connections of the orexin network in the regulation of arousal-related behavioral and endocrine responses [24,25]. Also, circulating peripheral or centrally released ghrelin significantly contributes to the hyperphagic activity of the orexins [89]. In efferentation, the orexin connectomics cooperates with the corticotrope-releasing hormone (CRH)–urocortin system [11], the network of NPY-positive [12,13] and monoaminergic [203] cells in the orchestration of the neuroendocrine responses to processed and homeostatic challenges [11,13]. Such interactions were established in other functions such as thermoregulation, mood, anxiety, learning [12,107,112,148,161,204,205], and reproduction [151]. Regarding arousal, one of the most important connections between the orexinergic system of the organism and the environment is established through the SCN [46]. The neuromedin-S released from the SCN of the hypothalamus might interpret these photic stimulations, which arrive at the SCN through the retinohypothalamic pathway [190]. Nevertheless, this aspect of hypocretin physiology must be further scrutinized and confirmed using experimental data.

During the investigation of pathophysiological alterations in the hypocretin/orexin network, some unique features of the system were unveiled. It is a well-known phenomenon in neuropeptide pathophysiology that the deficiency of a given neuropeptide or its secretory neurons usually does not bring about significant functional disturbances in the affected organism. This is due to the functional overlap between and redundancy of different neuropeptides or neuropeptide families. Typically, in congenital cases, during embryonic and fetal development, other neuropeptides can functionally compensate for the deficiency of the affected transcript even in knockout animals. Obviously, acquired abnormalities are less prone to correction due to the much more limited adaptation of the adult brain. Therefore, neuropeptide deficiencies do not cause such dramatic pathophysiological and clinical changes as is the case in congenital or acquired disorders of neurotransmission such as dopamine (phenylketonuria, Parkinson’s disease, and Sydenham’s chorea minor), GABA (Huntington’s disease and some forms of congenital epilepsies), or acetylcholine (myasthenia gravis, Lambert–Eaton myasthenic syndrome, and Alzheimer’s disease) metabolism disorders. However, the orexin/hypocretin system is different in this respect. Not only acquired but also congenital deficiency inevitably leads to severe pathophysiological changes, as exemplified in narcolepsy [70,71] or the blunted stress response exhibited by OX2R-deficient knockout mice [145]. This might be attributed to the fact that the orexin-positive neuron population does not exceed 50,000–80,000 cells in the hypothalamus [72], which makes it peculiarly sensitive to injuries. Furthermore, orexins bear weak structural resemblance only to a few members of the incretin family [23,24]. Even orexin-A and orexin-B differ in 50% of their primary structure, and they exhibit significantly different receptor affinities [23,24]. Therefore, it is not surprising that hardly any other neuronal network can take over the function of the orexinergic system. Some functional overlap might be provided by other GPCRs since certain neuropeptide receptors, such as the type-2 NPY receptor, the TRH receptor, the CCK type-A receptor, and the NK2 neurokinin receptor show some similarities (26%, 25%, 23%, and 20% identity, respectively) to the orexin receptors (OX1R and OX2R) [23]. However, their binding affinity to orexins is negligible [206]. The highest structural similarity is exhibited by the NPFF receptor of the RF-amide peptide family, which is 37% identical to OX1R and 35% identical to OX2R, respectively, and shows significant affinity to the orexins [35,36].

## 7. Aspects of Human Pathophysiology: The Present and Future Therapeutic Potential of Orexin Receptor Ligands

Even the first results of experiments carried out on the orexin/hypocretin system suggested that several human pathophysiological conditions could be explained by alterations in the orexin neurons [207]. Dysfunctions of the ARAS and sleep disorders, such as obstructive sleep apnea–hypopnea syndrome, were the first and somewhat obvious culprits, which were thoroughly and successfully investigated. Since then, both in narcolepsy with cataplexy [70,71,72,73,208] and in obstructive sleep apnea–hypopnea syndrome [209,210,211], the dysfunction of the orexin system has been established. Moreover, the acquired form of narcolepsy proved to be a classic example of a neuroinflammatory disorder. It seems to be evoked by H1N1 influenza virus infection or vaccination, which gives rise to an autoimmune reaction against the hypocretin neurons [74,212,213].

Increased tone of the orexinergic system, especially in cooperation with the ghrelin network, has also been suspected in disorders of the reward system. It appears that their synergistic hyperactivity is accountable for a rare form of monogenic obesity, Prader–Willi syndrome (PWS) [214]. However, the picture is more complex, as the orexin system is a double-edged sword; it increases feeding and energy expenditure simultaneously depending on the environmental cues. Accordingly, it has been implicated in both weight gain and weight loss [85], as well as in such disorders of food consumption as binge eating [215], bulimia, and anorexia nervosa [216]. Furthermore, in Kleine–Levin syndrome [217,218], the alternation of the alert and hyperphagic stages of hypersomnia has been connected to fluctuations in the orexin levels in the cerebrospinal fluid (CSF). It seems that, like the HPA response, the actual eating disorder is determined by the schedule and the modality of the psychological stressor [219], and it can manifest itself in seemingly opposite conditions.

Regarding reproductive processes, hypoactivity of the orexin system was observed in mothers suffering from gestational diabetes [220] and in patients diagnosed with polycystic ovary syndrome (PCOS) [221,222]. This might be related to the concomitant increase in the body weight and leptin levels of the patients, which downregulates orexin expression [223]. As for the pharmacological rewards, it is substance withdrawal that represents the common mechanism of orexin upregulation in different forms of drug addictions. Apparently, withdrawal symptoms are managed by individuals as stress stimuli, and they increase arousal, attention, and drug-seeking behavior [172,224,225].

In humans, the dysfunction of the orexin system in the regulation of the ARAS may also bring about the development of such diseases as attention deficit hyperactivity disorder (ADHD), anxiety, epilepsy, panic, and phobias [106,160,162,172,226]. As far as hyperactivity is concerned, the role of the orexin system has also been verified since the exaggerated startle response in anxious patients could be effectively reduced with an orexin receptor antagonist [227]. These conditions accompany the pathophysiological regulation of neuronal excitation and show clear circadian fluctuation, which reinforces the view that alteration in the orexin/hypocretin system plays a causative role in their development. Long periods of overexcitation have a detrimental impact on the neurons. In the burnt-out phase, these diseases give way to such chronic conditions as major depression, post-traumatic stress disorder (PTSD), psychosomatic problems like hypertension [228,229,230,231], and even neurodegenerative disorders [232]. Some further conditions such as cognitive disorders [233] and abnormal pain sensation [234,235,236] may also be related to alterations in alertness and the gating mechanism and therefore can be connected to the orexin/hypocretin system. However, it is important to emphasize that orexin receptors show some structural similarities to those of RF-amides [233]. Accordingly, their direct action may be mediated by not only their own receptors, expressed on the crucial gating elements (LC and PAG) of pain signaling, but also can be reinforced indirectly via the RF-amide receptors, which are expressed in both the ascending and descending pathways of pain sensation [237]. Abnormalities in orexin physiology have already been identified in chronic pain disorders, such as fibromyalgia [238], and especially in primary headaches such as migraine and cluster headaches [120,121,198]. The latter condition deserves special attention since these attacks show a clear diurnal pattern, and in its pathogenesis, the role of the SCN has already been verified [121]. According to the data from the literature, this analgesic action of the orexin network is mediated by orexin-A and OX1Rs [239].

The previously mentioned conformational overlap between the RF-amide and orexin receptors may also account for the reproductive and antineoplastic activities of orexins [28,240,241]. This is because RF-amides play a well-known role in the inhibition of metastasis formation [242], and in the past few years, they have emerged as metabolic regulators of the gonadal axis [243]. Since the activity of the RF-amide system shows clear periodicity, it is quite reasonable to imply that the orexin system may modulate its function either directly or indirectly [240].

Finally, it must be mentioned that the dysfunction of the orexin system was demonstrated in common neuropsychiatric, neuroinflammatory, and neurodegenerative disorders such as schizophrenia, Parkinson’s disease, Alzheimer’s disease, Huntington’s disease, multiple sclerosis (MS), and amyotrophic lateral sclerosis (ALS) [232,244,245,246,247]. Nevertheless, in these pathologic conditions, the dysfunction of the orexin system is not specific but can be attributed to the widespread devastation of the connectome. In these conditions, ultimately, all neural networks will be affected, but the orexin system is specifically frail and sensitive to focal injuries since it has a limited number of neurons, which are confined to a relatively small region. Therefore, it is among the first centers that succumb to the detrimental effects of misfolded protein aggregation and neuroinflammation. That is why some shared, conspicuous symptoms of the above-mentioned fatal disorders were identified as resulting from the failure of the orexin network. Cataplexy and dysfunction of the postural reflexes can be observed in Parkinson’s, Huntington’s, and prion diseases. Alterations in sleep patterns and vigilance are common findings in Alzheimer’s, MS, and prion diseases. Rapid fluctuations in mood, unwarranted anxiety, irrational fears, and extreme irritability are the common behavioral symptoms [248,249] in the above-mentioned diseases. Later, in all these symptom categories, either hyperactivity or hypoactivity of the orexin system has been suspected or already verified [232,244].

## 8. Promising Results in Translational Pharmacology

It is a well-known hindrance in neuropeptide pharmacology that often those compounds which possess the most promising biochemical features (affinity, activity, half-life, etc.) cannot bypass the blood–brain barrier (BBB) [7]. In several instances, only circumscribed areas (the lamina cribrosa or the circumventricular organs) provide access to the cerebrospinal fluid (CSF) to native ligands [7], or sophisticated nanocarriers (liposomes, nanoparticles, etc.) are required to surmount this pharmacokinetic obstacle [250]. However, in the case of the previously discussed feed-promoting neuropeptides such as ghrelin and the orexin system, both natural ligands and their chemically designed analogs can freely bypass the BBB [7,251,252]. Derivatives of orexins are especially successful in this regard because some of these antagonists have already been approved by the Food and Drug Administration (FDA) in the treatment of insomnia [113] (Table 2). Other antagonists, which are suggested for the treatment of panic, major depressive disorder, anxiety, and binge eating, are under investigation [113]. Studies on antagonists which are recommended for the treatment of narcolepsy are also in the clinical phase of pharmacological trials [113].

At present, the most coveted aim in neuroendocrine research is to engineer orexin derivatives which could relieve the abnormalities of the sleep–wake cycle in neurodegenerative disorders. They would be game-changers in palliative therapy, as traditional hypnotics are strong depressants and further deteriorate the function of the otherwise failing CNS. Therefore, present and future orexin derivatives are among the most pioneering and successful compounds in neuropeptide pharmacology and have huge potential in pharmaceutical development [113].

Additionally, emerging research explores the potential of non-invasive brain stimulation techniques (NIBS), such as transcranial magnetic stimulation (TMS) and transcranial direct current stimulation (tDCS). These NIBS techniques seem to be promising therapeutic alternatives as the orexin system occupies a well-circumscribed region in the CNS. Therefore, the symptoms of conditions like narcolepsy, cluster headaches, and affective and cognitive disorders that are associated with the dysfunction of the orexin system could be mitigated by them [262,263,264].

## 9. Discussion

Orexin/hypocretin neuropeptides are pivotal players in regulating various physiological processes, such as food intake, metabolism, the HPA axis, reproduction, and behavior [10,21,23,43,82,83,85,109,136,265,266]. They were primarily described to orchestrate such parameters of homeostatic balance as feeding, thermogenesis, and heat dissipation [12,23,43,85,97]. Later research shed light on their intricate involvement in the mediation and modulation of such behavioral paradigms as arousal, anxiety, fear, and the stress response [11,44,65,82,106,111,116,160]. The orexin system is also implicated in the regulation of pain sensation and the behavioral changes evoked by natural and pharmacological rewards such as addiction [79,106,117,120,181,198,224,267,268]. This way, dysfunctions in the orexin system have been associated with various human pathophysiological conditions, such as obesity, addictive disorders, narcolepsy, obstructive sleep apnea–hypopnea syndrome, anxiety, cognitive disorders, and abnormal mood fluctuations [10,109,122,215,227,230,269]. This review tries to seamlessly integrate the diverse activities of orexins and provides a more in-depth understanding of those fields such as stress response, fear, anxiety, and learning in which the authors have significantly contributed to the literature [11,12,13,107,112,161].

Regarding the limits of this article, it is important to note that the review is based on the existing literature and does not present any new experimental data. As a result, the caliber and scope of the studies included in the analysis limit the conclusions drawn from this review. Additionally, the review focuses on preclinical research on the orexin/hypocretin system, and the translation of these findings into clinical practice may be challenging. However, the review has several merits, including its interdisciplinary approach to understanding the orexin/hypocretin system, synthesizing information from various fields, including neuroscience, endocrinology, and pharmacology [10,21,83,85,109,113,270,271]. It could provide “food for thought” to researchers and clinicians interested in the orexin/hypocretin system, and it could inspire future research by identifying the knowledge gaps and areas that require further investigation.

The ultimate goal of the research on the orexin/hypocretin system is to develop effective therapeutic interventions for various disorders, such as sleep disorders, obesity, addiction, and anxiety [23,53,66,68,69,85,106,108,116,160,162,217,272,273,274,275]. However, this goal presents several challenges, including the need to understand the complex and multifaceted role of orexins in physiology and behavior, as well as the need to develop safe and effective drugs that target the orexin system. To achieve this goal, researchers need to have a deep understanding of the orexin/hypocretin system, including its molecular and cellular mechanisms, as well as its interactions with other systems in the body. They also need to develop advanced technologies for studying the orexin system, such as optogenetics, chemogenetics, and advanced imaging techniques. In addition, researchers need to develop safe and effective drugs that target selectively the orexin system, which requires a thorough understanding of the pharmacokinetics and pharmacodynamics of orexin derivatives. Overall, this line of research has the potential to improve the lives of millions of people worldwide, making it a crucial area of investigation, as is their potential therapeutic applications. Nevertheless, since several derivatives of orexins with high affinity and activity to their receptors can bypass the blood–brain barrier, some antagonists have already been approved by the FDA for the treatment of insomnia, and other antagonists and agonists are under investigation for the treatment of various disorders of food intake and behavior [113,271,276].

## 10. Conclusions

The interdisciplinary approach of this review has enhanced our understanding of the orexin/hypocretin neuropeptide family and its potential therapeutic applications. However, there are still several theoretical and methodological avenues that require refinement, such as the need for more precise and selective orexin receptor agonists and antagonists. Future research directions could focus on developing innovative drug delivery systems that can effectively target the orexin system while minimizing the off-target effects. Additionally, further research is needed to understand the complex interactions between the orexin system and other physiological and behavioral processes, such as the immune system and circadian rhythms. Overall, the orexin/hypocretin system is a fascinating area of research with significant theoretical and translational implications. By understanding the complex and multifaceted role of the orexin system, researchers can identify new drug targets and develop innovative drug delivery systems that can effectively treat various disorders. We hope that this review serves as a valuable resource for researchers and clinicians interested in the orexin/hypocretin system and the development of agents targeting this system.

## Figures and Tables

**Table 2:** Orexin analogs under clinical investigation [113,253].

Classes	Indications	Stage
OX2R agonists	Narcolepsy	Phase II. [254,255]
OX2R antagonists	Major depressive disorder (MDD)	Phase III. [256]
Dual antagonists	Insomnia	approved (e.g., Suvorexant [257,258], Lemborexant [259])
OX1R antagonists	Binge eating disorder	Phase II. [260]
	Panic disorder, MDD, anxiety	Phase II. [261]

## Data Availability

Data sharing is not applicable to this article.

## Abbreviations

ACTH	adrenocorticotropic hormone
ALS	amyotrophic lateral sclerosis
ARAS	ascending reticular activation system
ARC	arcuate nucleus
BAT	brown adipose tissue
BBB	blood–brain barrier
BNST	bed nucleus of stria terminalis
CeA	central amygdala
CCK	cholecystokinin
CNS	central nervous system
CSF	cerebrospinal fluid
CRH	corticotrope-releasing hormone
DMH	dorsomedial hypothalamus
DR	dorsal raphe
FDA	the Food and Drug Administration
GABA	γ-amino-butyric-acid
GAS	general adaptation syndrome
GPCRs	G-protein-coupled receptors
HPA	hypothalamic–pituitary–adrenal cortex
HPG	hypothalamic–pituitary–gonadal axis
IC	insular cortex
LA	lateral amygdala
LC	locus coeruleus
LDT	lateral dorsal tegmental nuclei
LHA	lateral hypothalamic area
MCH	melanin-concentrating hormone
MDD	major depressive disorder
MnPO	median preoptic nucleus
MPO	medial preoptic nucleus
MS	multiple sclerosis
MT	mesopontine tegmentum
MTL	medial temporal lobe
NAc	nucleus accumbens
NIBS	non-invasive brain stimulation techniques
NK	neurokinin
NM	neuromedin
NMS	neuromedin S
NPY	neuropeptide Y
NST	nucleus of the solitary tract
NT	neurotensin
OVLT	organum vasculosum laminae terminalis
OX1R	orexin-1 receptor
OX2R	orexin-2 receptor
OXR	orexin receptor
PAG	periaqueductal gray
PCOS	polycystic ovary syndrome
PFA	perifornical area
PFC	prefrontal cortex
POMC	pro-opiomelanocortin
PON	preoptic nucleus
PPT	pedunculopontine tegmental nucleus
PVN	paraventricular nuclei
RVLM	rostral ventrolateral medulla
RVMM	rostral ventromedial medulla
SA	sympathoadrenal
SCN	suprachiasmatic nucleus
SON	supraoptic nucleus
tDCS	transcranial direct current stimulation
TMs	transcranial magnetic stimulation
TMN	tuberomammillary nucleus
VMH	ventromedial hypothalamus
VTA	ventral tegmental area
VLPO	ventrolateral preoptic nucleus
